# DNA Demethylation in the Processes of Repair and Epigenetic Regulation Performed by 2-Ketoglutarate-Dependent DNA Dioxygenases

**DOI:** 10.3390/ijms221910540

**Published:** 2021-09-29

**Authors:** Nikita A. Kuznetsov, Lyubov Yu. Kanazhevskaya, Olga S. Fedorova

**Affiliations:** Institute of Chemical Biology and Fundamental Medicine Siberian Branch of Russian Academy of Sciences, 8 Prospekt Ak. Lavrentieva, 630090 Novosibirsk, Russia; Nikita.Kuznetsov@niboch.nsc.ru (N.A.K.); lyubov.kanazhevskaya@niboch.nsc.ru (L.Y.K.)

**Keywords:** DNA demethylation, DNA dioxygenase, oxygen activation, catalytic mechanism, direct repair, epigenetics

## Abstract

Site-specific DNA methylation plays an important role in epigenetic regulation of gene expression. Chemical methylation of DNA, including the formation of various methylated nitrogenous bases, leads to the formation of genotoxic modifications that impair DNA functions. Despite the fact that different pathways give rise to methyl groups in DNA, the main pathway for their removal is oxidative demethylation, which is catalyzed by nonheme Fe(II)/α-ketoglutarate–dependent DNA dioxygenases. DNA dioxygenases share a common catalytic mechanism of the oxidation of the alkyl groups on nitrogenous bases in nucleic acids. This review presents generalized data on the catalytic mechanism of action of DNA dioxygenases and on the participation of typical representatives of this superfamily, such as prokaryotic enzyme AlkB and eukaryotic enzymes ALKBH1–8 and TET1–3, in both processes of direct repair of alkylated DNA adducts and in the removal of an epigenetic mark (5-methylcytosine).

## 1. Introduction

Methylation is one of the most common chemical changes in nucleic acids. On the one hand, alkylating agents (extracellular or intracellular) can attack reactive groups in DNA and produce cytotoxic DNA lesions. On the other hand, site-directed enzymatic methylation of DNA plays an important signaling role in the epigenetic regulation of gene expression. Using S-adenosylmethionine as the most common electrophilic source of methyl groups in a living organisms, methyltransferases methylate DNA, thereby affecting the regulation of gene expression [1,2].

The functions of DNA methylation in the processes of DNA damage and in the regulation of DNA functions are well characterized. Demethylation, just as methylation, is also a key phenomenon in the regulation of the DNA methylome. Demethylation processes, in particular direct oxidative demethylation, lately have been attracting the attention of researchers owing to the complexity of the catalytic mechanism and specific features of the enzymes that catalyze this reaction.

**Figure 1:**
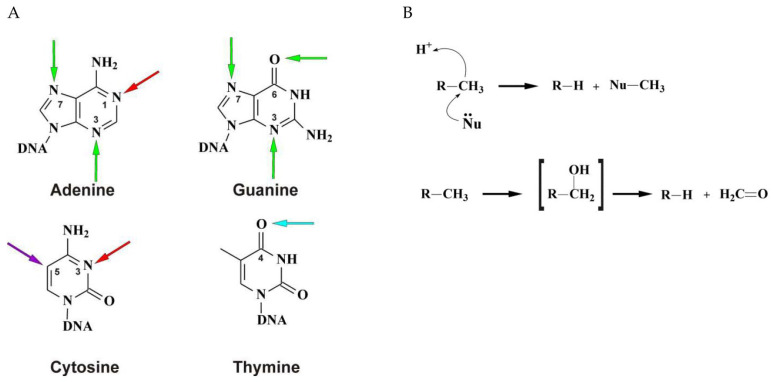
Methylation sites in nucleic acids. (**A**) Methylation positions in nitrogenous bases of a single- and double-stranded nucleic acid that are characteristic of the S_N_1 mechanism (blue), S_N_2 mechanism (red), or both types (green), epigenetic enzyme-catalyzed cytosine methylation (violet). (**B**) Schemes of demethylation via the mechanisms of nucleophilic substitution and oxidative demethylation.

In this review, we examine the enzymes involved in oxidative demethylation, which is mediated by the class of mononuclear Fe(II)-containing enzymes. Members of this class were discovered as DNA-repairing proteins that directly demethylate damaged DNA via the oxidation of an alkyl substituent. The DNA damage caused by methylating agents can occur at different positions of nitrogenous bases or of the 2′-deoxyribose-phosphate backbone. The outcome depends both on the type of chemical reaction (nucleophilic substitution S_N_1 or S_N_2) and on the nucleophilic center’s properties. In general, an S_N_1-type methylating agent (e.g., *N*-methyl-*N*′-nitrosourea) methylates an oxygen or nitrogen atom in nucleic acids, whereas S_N_2-type agents (e.g., methyl methanesulfonate) tend to attack open nucleophilic *N*-positions of the bases (Figure 1A) [3,4]. Various methylated lesions differ in the magnitude of cytotoxicity and mutagenic effects and can be promptly removed by enzymes through nucleophilic substitution or oxidative demethylation (Figure 1B) [3,5]. Only the DNA repair pathway proceeding via oxidative demethylation is discussed in this review.

*N*^1^-Methyladenine (m^1^A) and *N*^3^-methylcytosine (m^3^C) are the main lesions formed in single-stranded DNA under the action of S_N_2-type methylating agents [3,5]. The methylation of adenine or cytosine at these positions prevents the formation of Watson–Crick contacts between nitrogenous bases during DNA replication, thus having toxic effects on the cell [6]. Approximately 20 years ago, it was found that enzymes of the superfamily of Fe(II)/α-ketoglutarate (α-KG)-dependent dioxygenases are capable of removing methylation-related DNA damage via a unique mechanism of oxidative demethylation [7,8]. Since then, human orthologs of these enzymes have been found that they perform similar DNA repair functions. Moreover, studies on other proteins from this superfamily have revealed a number of demethylation functions that play an important part in the epigenetic methylation of histones, RNA, and DNA in higher eukaryotes. Recent research uncovered much more extensive and important involvement of this superfamily of enzymes in the control of gene expression through the demethylation of epigenetic DNA marks.

The enzymatic formation of 5-methylcytosine (m^5^C) is considered a marker modification of mammalian genomic DNA. Therefore, m^5^C is recognized as the “fifth nitrogenous base.” The modification of cytosine with the formation of m^5^C in CpG dinucleotide islands is one of the mechanisms of epigenetic regulation in mammalian cells [9,10,11]. The maintenance of the methylated state of certain genomic regions is important for the development of the cells, and anomalies in the “methylome” have negative consequences (for example, embryonic death [12] or carcinogenesis [13]). It was originally established in the 1960s that 2% to 8% of DNA is methylated in mammalian cells [14,15]. The process of DNA methylation is well understood, and much is known about molecular mechanisms of action of the participating enzymes, namely DNMT3a, DNMT3b (*de novo* methylation), and DNMT1 (maintenance of methylated sites) [15].

The reverse process, DNA demethylation, has been investigated in less detail. DNA demethylation can take place passively due to the “dilution” of methylated bases as a result of DNA replication [16,17]. Nevertheless, the rate of passive demethylation is not sufficient, for example, at the stages of early development of an organism (the zygote [18] and embryonic stem cells [19]), when at a certain point, there should be a sharp “reprogramming” and change of methylation sites between DNA replication rounds in the cell. Such phenomena obviously require an additional mechanism (i.e., active demethylation of DNA). In plants (for example, *Arabidopsis thaliana*), a class of m^5^C-DNA glycosylases has been found that removes m^5^C via base excision repair (BER) [15]. Although the enzymes that catalyze epigenetic DNA methylation in mammals have been characterized well [20], the enzymes responsible for demethylation have been unknown until the recent discovery of mononuclear nonheme Fe(II)-dependent dioxygenases of the TET (ten-eleven translocation) family [21]. It has been proven that the demethylation of the paternal genome in a fertilized egg [18,22], the removal of imprinting marks in embryonic cells [23], and the induction of tissue-specific genes [24] are mediated by active DNA demethylation.

Enzymes of the TET family can oxidize m^5^C to 5-hydroxymethylcytosine (hm^5^C), 5-formylcytosine (f^5^C), and then to 5-carboxylcytosine (ca^5^C) in a stepwise fashion. Subsequent removal of f^5^C and ca^5^C from DNA by BER is the first biochemically confirmed pathway of active demethylation in mammalian cells [25,26,27,28]. The TET family consists of three enzymes, TET1–TET3, which have a conserved catalytic site similar to the active site of AlkB and catalyze the sequential oxidation of a methyl group through the Fe(II)/α-KG–dependent mechanism.

## 2. The Catalytic Mechanism of Oxidative Demethylation

**Figure 2:**
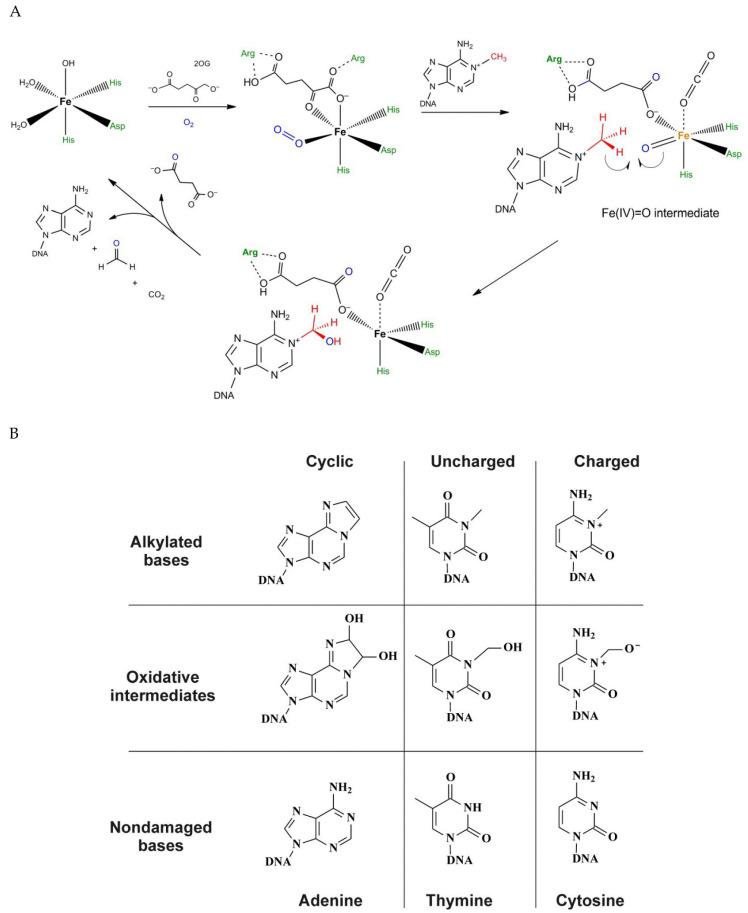
(**A**) The mechanism of catalytic oxidation of a methyl group by DNA dioxygenases as exemplified by *N*^1^-methyladenine (m^1^A) demethylation by prokaryotic DNA dioxygenase AlkB. (**B**) Oxidative repair of cyclic, uncharged, and charged alkylated bases [31].

Fe(II)/α-KG–dependent dioxygenases catalyze the direct oxidation of alkyl substituents in nitrogenous bases of DNA and RNA. The enzymes of this superfamily use nonheme iron as a cofactor and α-KG as a cosubstrate in the dealkylation reaction [29,30]. Figure 2A outlines the general mechanism of the catalytic reaction carried out by all Fe(II)/α-KG–dependent dioxygenases, with m^1^A demethylation by prokaryotic DNA dioxygenase AlkB as an example. At the first stage, molecular oxygen is added to the complex of the enzyme with the Fe(II) ion and α-KG. Next, the oxygen molecule is activated by the Fe(II) ion, as a result of which one of the oxygen atoms attacks α-KG, thereby causing its decomposition into succinate and CO_2_. Simultaneously, the iron ion and the remaining oxygen atom form a high-spin (S = 2) oxyferryl intermediate (Fe^IV^=O). At the second stage of the mechanism. This intermediate compound abstracts a hydrogen atom from the methyl group of the substrate with the formation of a Fe^III^–OH particle and a carbon radical in the DNA substrate. Eventually, the carbon radical withdraws the OH moiety from the Fe(II) coordination sphere, thus creating a hydroxylated intermediate which subsequently decomposes with the formation of adenine and formaldehyde.

With the help of mass spectrometry, the formation of epoxy intermediates has been registered during an interaction of AlkB with an exocyclic adduct (1,*N*^6^-ethenoadenine [εA] or 3,*N*^4^-ethenocytosine [εC]) [32,33], and the methods of in-crystal reactions have made it possible to detect glycol (resulting from εA), hemiaminal (from *N*^3^-methylthymine [m^3^T]), and a zwitterionic intermediate (from m^3^C) [31] (Figure 2B). The registration of the above intermediates confirms the mechanism of direct demethylation that includes the alkyl group oxidation. It has been shown that the charged zwitterionic intermediate contains a better leaving group than the neutral hemiaminal intermediate does. This observation explains the higher processing rate of AlkB toward m^1^A and m^3^C compared to m^3^T and *N*^1^-methylguanine [31].

In the absence of a substrate, the oxidation of iron and α-KG occurs too and, in this context, the formed oxo-complex of iron (IV) is capable of oxidizing the Trp178 side chain, resulting in irreversible modification of AlkB itself [34]. Furthermore, replacing the Fe(II) ion with Ni(II) or other metals eliminates the enzymatic activity. In addition, it is reported that analogs of α-KG, such as *N*-oxalylglycine, *N*-oxalylalanine, and 2-hydroxyglutarate, inhibit AlkB with high specificity [35,36,37].

## 3. Direct Demethylation of DNA and RNA by Prokaryotic and Eukaryotic DNA Dioxygenases of the AlkB Family

The removal of alkylated derivatives of nitrogenous bases from a nucleic acid can proceed via several pathways. Some of them are (1) the removal of bases by specific DNA glycosylases via BER pathway, (2) the removal of methylguanine by suicidal *O*^6^-methylguanine methyltransferases, and (3) direct removal of the methyl group by DNA dioxygenases of the AlkB family [6,38]. Fe(II)/α-KG–dependent dioxygenases from the AlkB family are the main repair enzymes that catalyze the direct removal of alkyl damage from nitrogenous bases of DNA and RNA. The AlkB dioxygenase is a participant of the adaptive response (Ada response) in *Escherichia coli*. Therefore, its expression level goes up significantly with an increase in the concentration of alkyl damage in cellular DNA [39].

**Figure 3:**
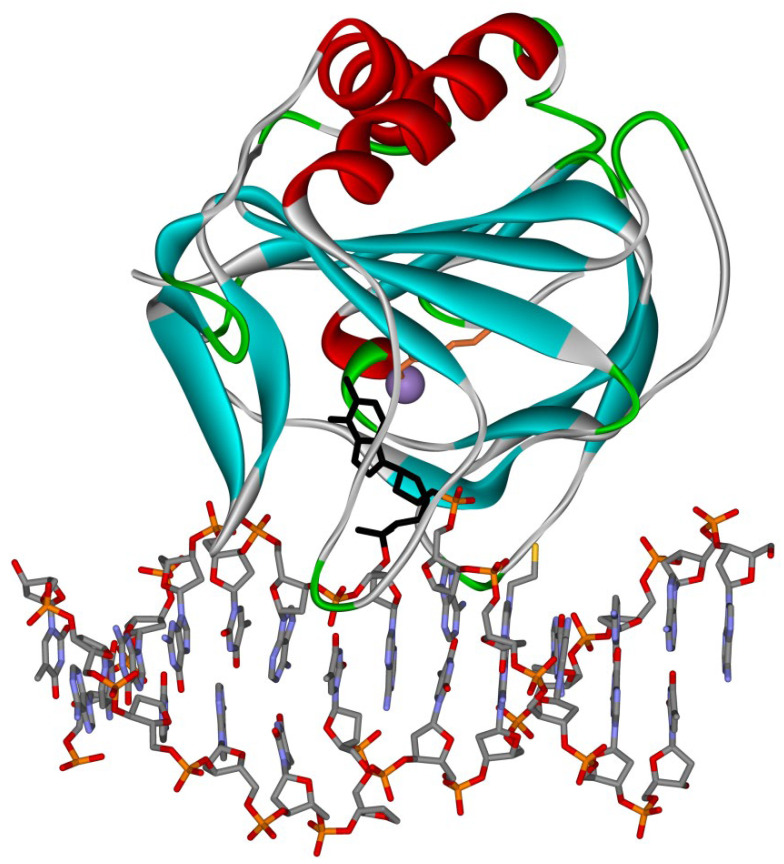
Three-dimensional structure of a complex of AlkB with DNA (Protein Data Bank ID: 3BIE). The figure shows damaged nucleotide m^1^A in the active site of the enzyme (black) and catalytic ion Mn(II) (purple).

All AlkB-like dioxygenases share a common structural topology (Figure 3) [40,41]. The active site of the enzyme is a part of the highly conserved double-stranded β-helix (DSBH) folded domain, which contains eight paired β-strands that form the so-called jelly-roll fold. The Fe(II) ion coordination in the fold of the DSBH domain is implemented by amino acid residues of the conserved His-X-Asp triad (where X is any amino acid). The feature that distinguishes AlkB family proteins from the large superfamily of Fe(II)/α-KG–dependent dioxygenases is the presence (in the DSBH domain) of a conserved arginine residue (Arg210 in AlkB), which is the main component of the substrate-binding pocket [42]. On the other hand, the structure of AlkB-like dioxygenases is rich in mobile loop regions, which are not conserved much. Supposedly, it is the length and amino acid composition of the loop regions that determine the broad substrate specificity of AlkB homologs and their ability to bind to single- and double-stranded nucleic acids [43].

There is a wide variety of homologs of the AlkB dioxygenase in mammalian cells, and the patterns of their subnuclear localization and substrate specificity differ considerably [44]. In particular, in human cells, nine orthologs of AlkB have been found, among which only the enzymes ALKBH1, ALKBH2, and ALKBH3 directly interact with DNA and RNA, and the others either engage in an interaction only with RNA (ALKBH5, ALKBH8, and ALKBH9 [also known as FTO]) or modify amino acid residues of protein targets (ALKBH4) [45]. To date, the potential cellular targets of dioxygenases ALKBH6 and ALKBH7 remain unclear. The wide substrate specificity of the enzymes from this family determines the diversity of dioxygenases’ functions in the cell. For instance, ALKBH2 is involved in the repair of ribosomal DNA [46], ALKBH3 repairs damage near replication forks [47], and ALKBH5 and ALKBH8 regulate gene expression by modifying mRNA and transfer RNA (tRNA) [48,49].

### 3.1. Prokaryotic DNA Dioxygenase AlkB

Even though the *alkB* gene in *E. coli* was discovered as far back as 1983 [50], the characterization of this gene took more than two decades. The *alkB* gene encodes a protein belonging to the Fe(II)/α-KG–dependent dioxygenase superfamily, which contains a wide variety of enzymes that catalyze the oxidation of various substrates by the oxygen molecule [51]. Early studies indicated that AlkB protects the cell from lethal effects of methyl methanesulfonate [52], and this process differed from the mechanisms of DNA repair known at the time [53]. It has also been shown that AlkB most likely prefers to act on single-stranded DNA and corrects m^1^A and m^3^C, which are the main products of methyl methanesulfonate’s action [53].

In 2002, two independent research groups proved that AlkB directly converts m^3^C and m^1^A into unmethylated bases by the mechanism of oxidative demethylation in the presence of Fe(II) ions, α-KG, and oxygen [7,8]. Since then, the data on the substrate specificity of AlkB have been expanded. It is known that this enzyme can repair such damaged bases as m^1^A, m^3^C, *N*^1^-methylguanine (m^1^G), m^3^T [54,55], exocyclic adducts (εA and εC) [32,56,57,58,59,60], and 3,*N*^4^-α-hydroxypropanocytosine [39] in single- and double-stranded DNA and RNA. All these lesions can be categorized into three groups: adducts positively charged at physiological pH, uncharged adducts, and cyclic adducts [31]. 

An analysis of the substrate specificity of AlkB has revealed that the enzyme predominantly repairs positively charged bases [39] since they bind best to the active site by interacting with negatively charged residue Asp135 [33,41]. In this case, the sequence of the polynucleotide is not essential for the catalysis, but for the efficient operation of AlkB, the presence of a 5′ phosphate group (in the oxidized nucleotide) is required [61], which binds to the positively charged groove composed of Thr51–Tyr55, Ser129, and Lys127. Amino acid residues Trp69 and His131 stabilize and hold the everted base in the active site owing to π–π stacking, while Tyr78, Lys134, Asp135, and Glu136 form specific hydrogen bonds with the modified nitrogenous base. At the N terminus, AlkB contains 90 unique amino acid residues, which form a flexible substrate-binding pocket that implements the binding of a wide range of modified bases [31,40,62].

AlkB preferentially oxidizes single-stranded DNA [63,64], indicating its association with the replication fork and a functional connection with transcription [65,66,67,68]. Additionally, this enzyme is active toward alkylated RNA in vitro and in vivo [68,69]. By means of an approach based on the formation of disulfide crosslinks, it has been found [41,70] that AlkB interacts almost exclusively with the strand containing damage and uses the mechanism of eversion of the modified nitrogenous base to create a catalytic complex, while the bases adjacent to the damage come closer together, thereby supporting stacking interactions. Therefore, the interaction with single-stranded DNA is more thermodynamically favorable since it does not imply disruption of Watson–Crick interactions in the base pairs adjacent to the damage.

### 3.2. ALKBH1

Human dioxygenase ALKBH1 has the highest degree of homology with bacterial dioxygenase AlkB despite containing a larger number of amino acid residues due to the presence of lengthy weakly ordered regions at the N and C termini [42]. Furthermore, the substrate specificity of ALKBH1 differs from that of its bacterial orthologue. For instance, ALKBH1 only weakly demethylates m^3^C in single-stranded DNA and is virtually inactive toward m^1^A in single- and double-stranded DNA [71]. On the other hand, this enzyme is involved in the demethylation of *N*^6^-methyladenine (m^6^A), which has been found in the genomic DNA of embryonic stem cells [72]. Besides, ALKBH1 can manifest AP-lyase activity toward apurinic/apyrimidinic sites in DNA [73]. Of note, in contrast to other homologs of AlkB, ALKBH1 is localized to mitochondria, not to the nucleus [71]. Haag et al. [74] have documented the ability of ALKBH1 to oxidize m^5^C to f^5^C in the methionine tRNA anticodon loop in mitochondria. This discovery points to possible participation of ALKBH1 in the regulation of translation in mitochondria since cells with a mutation in the *ALKBH1* gene are characterized by lower activity of translation and poor survival [74]. Another study has revealed the ability of ALKBH1 to methylate histone H2A in vitro and in vivo, thereby regulating its epigenetic status [75].

### 3.3. ALKBH2

Until recently, it has been thought that the ALKBH2 dioxygenase is only a repair enzyme that protects genomic DNA from nonbulky alkyl lesions such as m^1^A, m^3^C, m^3^T, εA, εC, and *N*^2,3^-ethenoguanine and does not interact with damaged RNA [76,77]. The difference in the substrate specificity between ALKBH2 and AlkB is explained by their low homology. As follows from X-ray data, an effective interaction of AlkB family dioxygenases with DNA and RNA strands is realized by characteristic loop regions, the so-called β-hairpins, whose size and amino acid composition differ substantially among homologues [40,41,64]. In particular, ALKBH2 contains two β-hairpins: a short hydrophobic hairpin containing the Phe102 residue and a long positively charged hairpin. The first one intercalates into the DNA duplex and occupies the space left from the damaged base flipped out into the active site, whereas the other one interacts with the intact DNA strand [41,78]. Apparently, the presence of the loop that is in contact with the complementary strand of the substrate determines the higher activity of ALKBH2 toward double-stranded DNA compared to single-stranded DNA. In addition, replacement of amino acid residues in the short hydrophobic hairpin with negatively charged ones significantly decreases the enzymatic activity toward double-stranded substrates but preserves the ability to bind single-stranded DNA [78]. The substrate specificity described above determines the main function of ALKBH2 in the cell: the repair of genomic DNA. Indeed, it has been demonstrated that this dioxygenase is located mainly in the nucleus, and during the S phase of the cell cycle, it is concentrated near replication forks. Here, ALKBH2 binds to the PCNA protein, which acts as a processivity factor for many replication and repair enzymes, and this property probably allows it to correct DNA damage within replication machinery [79].

A recent study revealed an ability of dioxygenase ALKBH2 to remove the epigenetic m^5^C mark from single- and double-stranded DNA in vitro [80]. Those investigators also analyzed the corresponding activity of dioxygenases AlkB and ALKBH3. The results indicated that ALKBH2 catalyzes the oxidation of m^5^C less efficiently as compared to the other dioxygenases and generates no more than 9% of the reaction product within the DNA duplex (~5% of the product within the single-stranded substrate). In the case of ALKBH2, the main product of the m^5^C transformation is hm^5^C, and the products of its further oxidation (f^5^C and ca^5^C) form only in negligible amounts. Biological significance of the discovered activity may be the implementation of a reserve pathway of epigenetic regulation in addition to the main mechanism, which is realized with the participation of TET family dioxygenases.

### 3.4. ALKBH3

Dioxygenase ALKBH3 is not only a structural but also functional homolog of AlkB from *E. coli*. In terms of substrate specificity, this enzyme is the closest to the bacterial one in comparison with other AlkB-like dioxygenases [66,68]. In particular, ALKBH3 works more efficiently with single-stranded DNA substrates and is capable of demethylating RNA nucleotides. At the same time, this dioxygenase virtually does not oxidize etheno derivatives of heterocyclic bases [81]. Since a complex of dioxygenase ALKBH3 with a DNA substrate has not yet been characterized by X-ray diffraction analysis, the question of which amino acid residues interact with single-stranded DNA remains open. It is believed that the specific loop of ALKBH2 that interacts with the intact strand of the DNA substrate and contains conserved triad Arg-Lys-Lys is practically absent in ALKBH3 [78]. Meanwhile, the active site cavity of the ALKBH3 is lined with side chain radicals of positively charged amino acids, making it more suitable for the binding to single-stranded DNA or single-stranded RNA substrates. In general, the efficiency of demethylation of such lesions as m^1^A and m^3^C by ALKBH3 is 2.6–4.0-fold lower than the efficiency of their transformation by ALKBH2 (in terms of the k_cat_/K_m_ ratio) [82]. These data, as well as the capacity of ALKBH3 to restore mRNA and tRNA nucleotides after treatment with methyl methanesulfonate (as shown in ref. [69]), are suggestive of a role of this dioxygenase in RNA repair during translation. This assumption is supported by the finding that ALKBH3 knockout mice do not experience an increase in the amount of m^1^A in their genomic DNA as compared to wild-type mice [83]. On the other hand, there is active research into the involvement of ALKBH3 in the repair of single-stranded DNA fragments produced by helicases and recombinases. For example, it is reported that in vitro and in vivo, this enzyme is capable of binding to helicase ASCC3 from the activating signal cointegrator complex (ASCC), which generates single-stranded DNA fragments [47]. The demethylating activity of the ALKBH3–ASCC3 complex toward m^3^C is significantly higher in comparison with the free dioxygenase. Furthermore, there are documented protein–protein interactions between ALKBH3 and recombinase RAD51C, which functions in homologous recombination of DNA ends during the repair of double-strand breaks [83]. The addition of RAD51C to ALKBH3 increases the efficiency of demethylation of m^3^C-containing DNA by 2.6-fold, suggesting that ALKBH3 takes part in repair in the vicinity of replication forks.

At present, the involvement of dioxygenase ALKBH3 in the regulation of gene expression is being actively investigated. Some authors [80] have published the proof of principle for the oxidation of the epigenetic m^5^C mark by ALKBH3. In this context, for a double-stranded DNA substrate, the predominant oxidation products are hm^5^C and f^5^C, and in the case of a single-stranded substrate, there is also ca^5^C. The overall m^5^C-oxidative activity of ALKBH3 is comparable in magnitude to that of AlkB and significantly exceeds the activity of ALKBH2.

### 3.5. ALKBH5, ALKBH8, and ALKBH9 (FTO)

Some AlkB-like dioxygenases have acquired the ability to demethylate RNA during evolution. The main alkyl modifications of ribonucleotides are m^1^A and m^6^A, the former being a lesion, and the latter an epigenetic mark [84]. Additionally, in the tRNA sequence of eubacteria, eukaryotes, and archaea, there are *N*^7^-methylated guanine residues, which stabilize the RNA’s secondary structure. The latest data indicate that m^6^A is most often located in a specific nucleotide context near stop codons and 3′ untranslated regions of mRNA and is a major epigenetic mark [85]. In human cells, the removal of the methyl group from m^6^A is performed by the ALKBH5 dioxygenase, and the structure of its DSBH domain slightly differs from that of its homologs [86]. Due to the greater distance between the DNA-binding loops, the ALKBH5 active-site pocket has a more open conformation. The disulfide bridge between Cys230 and Cys267, which is unique for dioxygenases of the AlkB family, apparently participates in the discrimination between single- and double-stranded substrates and keeps m^6^A in an everted state within the active site. The expression level and activity of dioxygenase ALKBH5 can vary greatly during carcinogenesis, implying its key role not only in the regulation of mRNA translation and RNA metabolism but also in hypoxia and autophagy [87]. Another dioxygenase that catalyzes the removal of the m^6^A methyl group from RNA is obesity-associated protein FTO also known as ALKBH9. A distinctive feature of FTO structure is the presence of two positively charged lysine-rich loops that have high affinity for the sugar-phosphate backbone and, just as with tweezers, hold the RNA substrate [88]. Until recently, it has been thought that the main substrate of FTO is m^6^A-containing RNA regions, but a recent study [89] revealed a high activity of this dioxygenase toward *N*6,2′-*O*-dimethyladenine that is located at the 5′ end of mRNA and is linked to 7-methylguanine via a triphosphate spacer [89]. This structure stabilizes eukaryotic mRNA.

Another RNA-specific human homolog of AlkB is dioxygenase ALKBH8, which is active toward methylated uridine in the tRNA anticodon loop [90]. This enzyme has a two-domain structure that includes an S-adenosylmethionine–dependent methyltransferase domain and AlkB-like domain [91]. During catalysis, using the methylase domain, ALKBH8 modifies 5-carboxymethyluridine (cm^5^U) into 5-methoxycarbonylmethyluridine, which is further hydroxylated into 5S-methoxycarbonyl-hydroxymethyluridine by means of the DSBH domain. Such a substrate specificity makes this dioxygenase more similar to hydroxylases of the TET family.

### 3.6. ALKBH4, ALKBH6, and ALKBH7

Other dioxygenases from the AlkB family share high homology of the DSBH domain but use proteins rather than nucleic acids as substrates. For dioxygenase ALKBH4, no crystal structure has been published so far, but electron paramagnetic resonance spectroscopy has confirmed the presence of a conserved iron-binding site, and an ability to oxidize the α-KG cosubstrate to succinate has been shown, reliably indicating that ALKBH4 belongs to the AlkB family [92]. For a long time, the substrate of this enzyme has been unknown, until Lee et al. [93] demonstrated the capacity of ALKBH4 for actin demethylation in the actomyosin complex during cytokinesis. Those authors [93] hypothesized that acetylated aspartate residues are the targets of oxidative demethylation in this case, as are methylated histidine and lysine residues. The substrate specificity of proteins ALKBH6 and ALKBH7 has not yet been clearly characterized. The data from a crystallographic analysis of ALKBH7 point to the absence of a DNA-binding motif in this protein. Therefore, this dioxygenase most likely does not interact with nucleic acids. So far, the least studied protein is the ALKBH6 dioxygenase, for which 3D structure has not been determined. In a recently published work, the Kang group [94] demonstrated the ability of an ALKBH6 ortholog from *A. thaliana* to bind single-stranded RNA. However, this enzyme was found to bind intact RNA and m^1^A-, m^3^C-, and m^6^A-containing RNA with the same efficiency, indicating low selectivity of ALKBH6 toward damaged bases. Consequently, further research is needed to identify the structure of substrates and biological functions of these dioxygenases.

## 4. Epigenetic Demethylation of DNA

The DNA methylation producing m^5^C is the most typical epigenetic modification and plays an important part in the processes of genomic marking, gene expression regulation, and development in mammals [18,20,95]. A methylation pattern is established by DNA methyltransferases (DNMTs), namely by enzymes DNMT3A and DNMT3B [96,97], and is then maintained by DNMT1 methyltransferase activity during DNA replication [98]. It should be noted that the dynamic regulation of various processes via the formation of m^5^C should involve the reverse phenomenon, demethylation. Even though the enzymes that catalyze DNA methylation have been characterized well, demethylation in mammals had remained poorly understood until the discovery of DNA dioxygenases of the TET family [25,99].

Enzymes of the TET family (TET1–TET3) catalyze the sequential oxidation of m^5^C to hm^5^C, f^5^C, and ca^5^C [25,26,27,28]. In vitro, all TET enzymes generate the final reaction product, ca^5^C, although TET2 prefers oxidizing m^5^C over the oxidation of hm^5^C and f^5^C [100,101].

**Figure 4:**
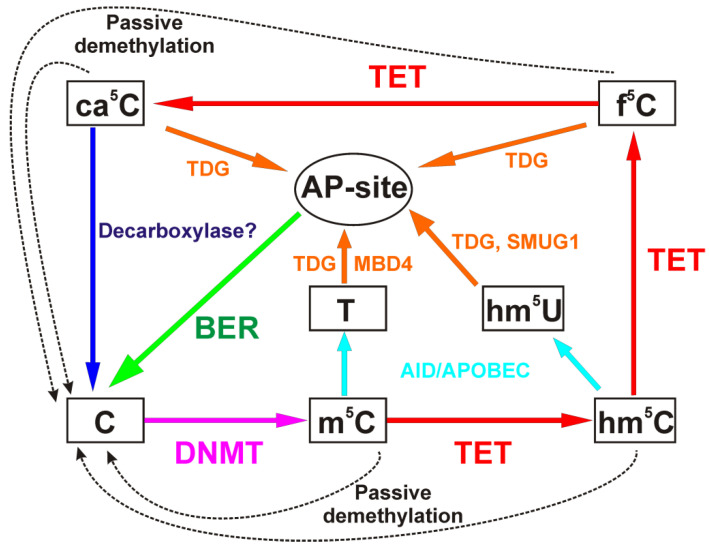
A diagram of active and passive cytosine demethylation pathways during epigenetic regulation. m^5^C residues are deaminated and oxidized thereby yielding uracil derivatives and oxidized m^5^C derivatives, which are removed by DNA glycosylases TDG, SMUG1, and MBD4 in BER [115].

In comparison with demethylation at a nitrogen position, the removal of the methyl group from a carbon atom is a much more complicated process since the C–C bond is very inert under physiological conditions. For this reason, all of the oxidized derivatives of m^5^C, namely hm^5^C, f^5^C, and ca^5^C, are quite stable under normal conditions within the cell. Since the oxidized derivatives of m^5^C are more hydrophilic, m^5^C oxidation can be functionally regarded as “demethylation.” Nevertheless, the complete removal of the modified nitrogenous base requires the participation of DNA glycosylases, which specifically recognize the modified nucleotide and initiate BER [102,103] (Figure 4). Of note, DNA molecules containing hm^5^C in CpG dinucleotides are very poor substrates for DNMT1 [104,105] since DNMT1 forms an unproductive complex with DNA duplexes containing oxidized forms of m^5^C [106]. This means that after the oxidation of m^5^C to hm^5^C, DNA methylation can no longer be preserved even in the presence of DNMT1.

Accordingly, in addition to passive demethylation, i.e., when oxidized cytosine derivatives are “diluted” during replication, the existence of a replication-independent mechanism of active demethylation has been proposed. It has been demonstrated that f^5^C and ca^5^C are recognized and removed from DNA with the formation of an apurinic/apyrimidinic site by thymine DNA glycosylase TDG [26,107,108]. hm^5^C can accumulate to levels on the order of 20–30% of m^5^C levels, in particular in mammalian neurons, whereas f^5^C and ca^5^C are present in most tissues only in negligible amounts [100,109,110,111].

It must be pointed out that after the first catalytic cycle of m^5^C oxidation, hm^5^C is formed, which can be deaminated by enzymes of the AID/APOBEC family to 5-hydroxymethyluracil (hm^5^U). This base is removed from DNA by either TDG or SMUG1 (single-stranded monofunctional uracil-DNA glycosylase 1), and in this way, the BER pathway is initiated too [112]. Nonetheless, this mechanism is still debated since it has been reported that purified deaminases AID/APOBECs are unable to catalyze the conversion of modified cytosines in vitro [113]. As another pathway for the removal of m^5^C conversion products, the involvement of decarboxylase for the direct conversion of ca^5^C to cytosine has been theorized but not yet confirmed experimentally [114].

### 4.1. Structural Features of TET Family Enzymes

The structure of the TET family proteins is rather conserved; all TET enzymes contain a Cys-rich region and a DSBH sequence characteristic of dioxygenases that consists of two β-sheets and serves for the binding of an Fe(II) ion and α-KG and for catalytic site assembly [25]. Besides, TET1 and TET3 contain a binding site for unmethylated CpG domains (CXXC) [116].

Currently, the various functions, various genomic activities, and distributions of different isoforms of the TET family enzymes are unclear. Nevertheless, it can be theorized [117] that one of the main functions of these proteins is the epigenetic repair that maintains the unmethylated state of CpG islands. This model is supported by evidence that TET1 and TET3 are strongly specific to unmethylated CpG islands, probably due to CXXC zinc finger domains [118,119,120,121]. On the contrary, TET2 mainly takes part in the binding and demethylation of enhancers [122]. The TET2 enzyme does not contain a CXXC domain but forms a functional heterodimer with the CXXC4 protein, which can provide this domain to TET2 [123].

**Figure 5:**
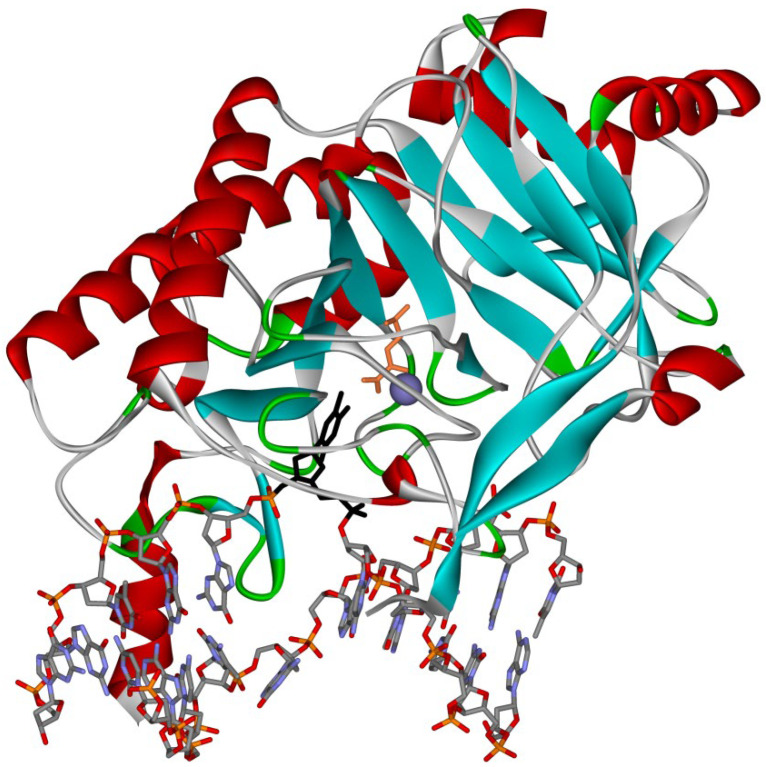
Three-dimensional structure of a TET2–DNA complex (Protein Data Bank ID: 4NM6). The figure shows damaged nucleotide m^5^C in the active site of the enzyme (black) and catalytic ion Fe(II) (purple).

DSBH and Cys domains are required for the catalytic activity of TET. According to data from an X-ray diffraction analysis [124], the Cys-rich region “is wrapped” around the DSBH domain and is a part of the catalytic site (Figure 5). A DNA duplex makes contacts with two loops of the C-terminal part of the Cys-rich domain. In this context, only the m^5^CpG dinucleotide and the sugar-phosphate backbone of DNA are engaged in direct contacts with the enzyme, consistently with a finding that the DNA context does not affect the selectivity of this oxidation reaction in vitro [124].

### 4.2. Substrate Specificity

Although DNA and RNA methylation are performed by distinct methyltransferases [125], TET enzymes are involved in the oxidation of both DNA and RNA. It is reported that all three TET enzymes are active toward 5-methyl-ribocytosine (m^5^rC) in vitro and in transfected cells [126], and furthermore, the reactivity is higher toward single-stranded DNA substrates than single-stranded RNA substrates [126]. There is evidence [127] of a relation between the nature of the target nucleotide and TET reactivity. The decrease in reactivity toward single-stranded RNA can be reversed by removing 2′-OH from the target nucleotide. Accordingly, the addition of 2′-OH to a target nucleotide reduces the activity toward single-stranded DNA to the level observed with the single-stranded RNA substrate. A molecular dynamics simulation has revealed that m^5^rC fits into the active site of TET2. Even though an m^5^rC substrate can be sterically and conformationally accommodated by the active site, the interaction with m^5^rC is energetically unfavorable as compared to m^5^C.

An analysis of X-ray structural data [101] suggests that pyrimidine bases m^5^C, hm^5^C, and f^5^C assume almost identical conformations in the active-site pocket. The main differences are related to the arrangement of cytosine substituents; for instance, the hydrophobic methyl group of m^5^C is directly pointed at the catalytic site and is not in contact with residues of α-KG or TET2. By contrast, the hydroxymethyl group of hm^5^C adopts a hindered conformation by forming a hydrogen bond (~2.6 Å) with α-KG 1-carboxylate, whereas the formyl group of f^5^C is hindered by the intramolecular hydrogen bond between the carbonyl group and exocyclic nitrogen atom *N*^4^ of cytosine.

Therefore, the conformational differences among m^5^C, hm^5^C, and f^5^C in these complexes are a consequence of their derivatives’ properties. It is these differences that can influence the efficiency of oxidation of m^5^C, hm^5^C, and f^5^C during the enzymatic reaction in question.

Some researchers have proposed a consensus mechanism of action of Fe(II)/α-KG–dependent dioxygenases, which includes four reaction stages (Figure 2A). In this mechanism, hydrogen atom abstraction is the rate-limiting step of oxidation, as in the case of AlkB [128]. To test whether the hydrogen abstraction is a stage of TET2-mediated oxidation of a substrate, the catalytic activity of TET2 has been determined toward a DNA substrate containing deuterated m^5^C in which all the hydrogen atoms of the methyl group were replaced by deuterium [128]. The results showed that the hydrogen abstraction is also a key step in the TET2-mediated oxidation of m^5^C.

## 5. Cross-Specificity of TET and AlkB Enzymes

Assays of substrate specificity suggest that TET enzymes are active not only toward m^5^C but also toward other lesions in DNA and RNA as well as toward alternative cytosine residues modified at position 5, such as 5-ethylcytosine [126,127,129,130,131] (Table 1). In an investigation into the factors that determine m^5^C recognition among other bases, it was recently established [132] that TET enzymes can also act as direct N-demethylases of cytosine bases. It was demonstrated that the TET enzymes oxidize m^5^C with equal efficiency and participate in the direct demethylation of cytosine carrying *N*^4^-methyl substituents. The results showed that the TET enzymes have high plasticity of the active site, possibly indicating the existence of as yet unknown substrates that can reveal new biological functions of TET family members.

Of note, there is recent evidence for the ability of AlkB family enzymes—including human proteins ALKBH2 and ALKBH3 and their bacterial ortholog from *E. coli* AlkB—to oxidize the epigenetic modulator m^5^C to hm^5^C, f^5^C, and ca^5^C in vitro [80]. Given that both lesions m^5^C and m^3^C contain a methyl group located on opposite sides of the pyrimidine ring, the authors of [80] hypothesized that a switch of the *anti*-conformation of the glycosidic bond (m^3^C) to the *syn*-conformation (m^5^C) would make it possible to place the nitrogenous base in the active site with the correct arrangement of the methyl groups relative to catalytic amino acid residues and a cofactor. This hypothesis was proven by in vitro experiments, which showed that AlkB enzymes can modify the m^5^C epigenetic mark and generate its oxidized derivatives. Thus, the ability to oxidize the methyl group attached to a carbon atom rather than nitrogen in a nitrogenous base of DNA was demonstrated for the first time for AlkB family enzymes.

## 6. Conclusions

Despite chemical and enzymatic DNA methylation are well characterized processes, DNA demethylation, which play an important role both in DNA repair and regulation of DNA functions by epigenetic signaling, proceeds in the complex enzymatic pathway. Direct DNA demethylation catalyzed by mononuclear nonheme Fe(II)-dependent dioxygenases proceeds via the oxidation of an alkyl substituent. Nine eukaryotic DNA dioxygenases, ALKBH1-ALKBH9, as well as three enzymes, TET1–TET3, which have a conserved catalytic site similar to the active site of the prokaryotic enzyme AlkB, catalyze the sequential oxidation of an alkyl group through the Fe(II)/α-KG–dependent mechanism. In this review, generalized data on the substrate specificity and the catalytic mechanism of action of DNA dioxygenases of two different families AlkB and TET were analyzed. It was believed that the substrate specificity of the enzymes from these families was completely different. However recently it was discovered [80] that ALKBH2 and ALKBH3 catalyzed oxidative demethylation of the epigenetic marker m^5^C.

Given that m^5^C formation and demethylation are important epigenetic processes, a living organism can employ several redundant or complementary pathways to control this modification. Therefore, the oxidative demethylation of m^5^C by enzymes ALKBH2 and ALKBH3 is most likely an auxiliary pathway for this biological function. To sum up, AlkB proteins not only can repair such adducts as m^3^C, m^3^T, and other alkylated derivatives of nitrogenous bases in DNA but also are able to edit epigenetic modifications of m^5^C and generate the corresponding oxidized derivatives. These data indicate a strong connection between DNA repair and epigenetic modification of genes.

## Figures and Tables

**Table 1:** A summary of 2-ketoglutarate-dependent DNA dioxygenases and their alkylated DNA and RNA substrates.

Alkylated Base	Dioxygenase
m^1^A	AlkB, ALKBH2, ALKBH3
m^3^C	AlkB, ALKBH1, ALKBH2, ALKBH3
m^1^G	AlkB, ALKBH1, ALKBH3
m^3^T	AlkB, ALKBH2, ALKBH3, FTO
m^6^A	AlkB, ALKBH5, FTO
εA	AlkB, ALKBH2
εC	AlkB, ALKBH3
m^4^C	AlkB, TET1, TET2
m^5^C	TET1, TET2, TET3, AlkB, ALKBH1, ALKBH2, ALKBH3
hm^5^C	TET1, TET2, TET3, AlkB, ALKBH2, ALKBH3
f^5^C	TET1, TET2, TET3, AlkB, ALKBH2, ALKBH3
cm^5^U	ALKBH8
m^5^rC	TET1, TET2, TET3
